# Open Data, Open Source and Open Standards in chemistry: The Blue Obelisk five years on

**DOI:** 10.1186/1758-2946-3-37

**Published:** 2011-10-14

**Authors:** Noel M O'Boyle, Rajarshi Guha, Egon L Willighagen, Samuel E Adams, Jonathan Alvarsson, Jean-Claude Bradley, Igor V Filippov, Robert M Hanson, Marcus D Hanwell, Geoffrey R Hutchison, Craig A James, Nina Jeliazkova, Andrew SID Lang, Karol M Langner, David C Lonie, Daniel M Lowe, Jérôme Pansanel, Dmitry Pavlov, Ola Spjuth, Christoph Steinbeck, Adam L Tenderholt, Kevin J Theisen, Peter Murray-Rust

**Affiliations:** 1 Analytical and Biological Chemistry Research Facility, Cavanagh Pharmacy Building, University College Cork, College Road, Cork, Co. Cork, Ireland; 2 NIH Center for Translational Therapeutics, 9800 Medical Center Drive, Rockville, MD 20878, USA; 3 Division of Molecular Toxicology, Institute of Environmental Medicine, Nobels väg 13, Karolinska Institutet, 171 77 Stockholm, Sweden; 4 Unilever Centre for Molecular Sciences Informatics, Department of Chemistry, University of Cambridge, Lensfield Road, CB2 1EW, UK; 5 Department of Pharmaceutical Biosciences, Uppsala University, Box 591, 751 24 Uppsala, Sweden; 6 Department of Chemistry, Drexel University, 32nd and Chestnut streets, Philadelphia, PA 19104, USA; 7 Chemical Biology Laboratory, Basic Research Program, SAIC-Frederick, Inc., NCI-Frederick, Frederick, MD 21702, USA; 8 St. Olaf College, 1520 St. Olaf Ave., Northfield, MN 55057, USA; 9 Kitware, Inc., 28 Corporate Drive, Clifton Park, NY 12065, USA; 10 Department of Chemistry, University of Pittsburgh, 219 Parkman Avenue, Pittsburgh, PA 15260, USA; 11 eMolecules Inc., 380 Stevens Ave., Solana Beach, California 92075, USA; 12 Ideaconsult Ltd., 4.A.Kanchev str., Sofia 1000, Bulgaria; 13 Department of Engineering, Computer Science, Physics, and Mathematics, Oral Roberts University, 7777 S. Lewis Ave. Tulsa, OK 74171, USA; 14 Leiden Institute of Chemistry, Leiden University, Einsteinweg 55, 2333 CC Leiden, The Netherlands; 15 Department of Chemistry, State University of New York at Buffalo, Buffalo, NY 14260-3000, USA; 16 Université de Strasbourg, IPHC, CNRS, UMR7178, 23 rue du Loess 67037, Strasbourg, France; 17 GGA Software Services LLC, 41 Nab. Chernoi rechki 194342, Saint Petersburg, Russia; 18 Cheminformatics and Metabolism Team, European Bioinformatics Institute (EBI), Wellcome Trust Genome Campus, Hinxton, Cambridge, UK; 19 Department of Chemistry, University of Washington, Seattle, WA 98195, USA; 20 iChemLabs, 200 Centennial Ave., Suite 200, Piscataway, NJ 08854, USA

## Abstract

**Background:**

The Blue Obelisk movement was established in 2005 as a response to the lack of Open Data, Open Standards and Open Source (ODOSOS) in chemistry. It aims to make it easier to carry out chemistry research by promoting interoperability between chemistry software, encouraging cooperation between Open Source developers, and developing community resources and Open Standards.

**Results:**

This contribution looks back on the work carried out by the Blue Obelisk in the past 5 years and surveys progress and remaining challenges in the areas of Open Data, Open Standards, and Open Source in chemistry.

**Conclusions:**

We show that the Blue Obelisk has been very successful in bringing together researchers and developers with common interests in ODOSOS, leading to development of many useful resources freely available to the chemistry community.

## Background

The Blue Obelisk movement was established in 2005 at the 229^th ^National Meeting of the American Chemistry Society as a response to the lack of Open Data, Open Standards and Open Source (ODOSOS) in chemistry. While other scientific disciplines such as physics, biology and astronomy (to name a few) were embracing new ways of doing science and reaping the benefits of community efforts, there was little if any innovation in the field of chemistry and scientific progress was actively hampered by the lack of access to data and tools. Since 2005 it has become evident that a good amount of development in open chemical information is driven by the demands of neighbouring scientific fields. In many areas in biology, for example, the importance of small molecules and their interactions and reactions in biological systems has been realised. In fact, one of the first free and open databases and ontologies of small molecules was created as a resource about chemical structure and nomenclature by biologists [1].

The formation of the Blue Obelisk group is somewhat unusual in that it is not a funded network, nor does it follow the industry consortium model. Rather it is a grassroots organisation, catalysed by an initial core of interested scientists, but with membership open to all who share one or more of the goals of the group:

**• Open Data in Chemistry**. One can obtain all scientific data in the public domain when wanted and reuse it for whatever purpose.

**• Open Standards in Chemistry**. One can find visible community mechanisms for protocols and communicating information. The mechanisms for creating and maintaining these standards cover a wide spectrum of human organisations, including various degrees of consent.

**• Open Source in Chemistry**. One can use other people's code without further permission, including changing it for one's own use and distributing it again.

Note that while some may advocate also for Open Access to publications, the Blue Obelisk goals (ODOSOS) focus more on the availability of the underlying scientific data, standards (to exchange data), and code (to reproduce results). All three of these goals stem from the fundamental tenants of the scientific method for data sharing and reproducibility.

The Blue Obelisk was first described in the CDK News [2] and later as a formal paper by Guha et al. [3] in 2006. Its home on the web is at http://blueobelisk.org. This contribution looks back on the work carried out by the Blue Obelisk over the past 5 years in the areas of Open Data, Open Source, and Open Standards in chemistry.

## Scope

The Blue Obelisk covers many areas of chemistry and chemical resources used by neighbouring disciplines (*e.g*. biochemistry, materials science). Many of the efforts relate to cheminformatics (the scope of this journal) and we believe that many of the publications in Journal of Cheminformatics could be completely carried out using Blue Obelisk resources and other Open Source chemical tools. The importance of this is that for the first time it would allow reviewers, editors and readers to validate assertions in the journal and also to re-run and re-analyse parts of the calculation.

However, Blue Obelisk software and data is also used outside cheminformatics and certainly in the five main areas that, for example, Chemical Markup Language (CML) [4] supports:

1. **Molecules: **This is probably the largest area for Blue Obelisk software and data, and is reflected by many programs that visualise, transform, convert formats and calculate properties. It is almost certain that any file format currently in use can be processed by Blue Obelisk software and that properties can be calculated for most (organic compounds).

2. **Reactions: **Blue Obelisk software can describe the semantics of reactions and provide atom-atom matching and analyse stoichiometric balance in reactions.

3. **Computational chemistry: **Blue Obelisk software can interpret many of the current output files from calculations and create input for jobs. The Quixote project (see below and elsewhere in this issue) shows that Open Source approaches based on Blue Obelisk resources and principles are increasing the availability and re-usability of computational chemistry.

4. **Spectra: **1-D spectra (NMR, IR, UV etc.) are fully supported in Blue Obelisk offerings for conversion and display. There is a limited amount of spectral analysis but the software gives a platform on which it should be straightforward to develop spectral annotation and manipulation. However, currently the Blue Obelisk lacks support for multi-dimensional NMR and multi-equipment spectra (*e.g*. GC-MS).

5. **Crystallography: **The Blue Obelisk software supports the bi-directional processing of crystal structure files (CIF) and also solid-state calculations such as plane-waves with periodic boundary conditions. There is considerable support for the visualisation of both periodic and aperiodic condensed objects.

Many of the current operations in installing and running chemical computations and using the data are integration and customisation rather than fundamental algorithms. It is very difficult to create universal platforms that can be distributed and run by a wide range of different users, and in general, the Blue Obelisk deliberately does not address these. Our approach is to produce components that can be embedded in many environments, from stand-alone applications to web applications, databases and workflows. We believe that a chemical laboratory with reasonable access to common software engineering techniques should be able to build customised applications using Blue Obelisk components and standard infrastructure such as workflows and databases. Where the Blue Obelisk itself produces data resources they are normally done with Open components so that the community can, if necessary, replicate them. 

Much of the impetus behind Blue Obelisk software is to create an environment for chemical computation (including cheminformatics) where all of the components, data, specifications, semantics, ontology and software are Openly visible and discussable. The largest current uses by the general chemical community are in authoring, visualisation and cheminformatics calculations but we anticipate that this will shortly extend into mainstream computational chemistry and solid-state. Although many of the authors are employed as research scientists, there are also several people who contribute in their spare time and we anticipate an increasing value and use of the Blue Obelisk in education at all levels.

## Open Source

The development of Open Source software has been one of the most successful of the Blue Obelisk's activities. The following sections describe recent work in this area, and Table 1 provides an overview of the projects discussed and where to find them online.

**Table 1:** Blue Obelisk Open Source Software projects discussed in the text

Name	Website
**CML Tools**	

**CMLXOM**	https://bitbucket.org/wwmm/cmlxom/

**JUMBO**	http://sourceforge.net/projects/cml/

**Cheminformatics Toolkits**	

**Chemistry Development Kit (CDK)**	http://cdk.sf.net

**Cinfony**	http://cinfony.googlecode.com

**Indigo**	http://ggasoftware.com/opensource/indigo

**JOELib**	http://sf.net/projects/joelib

**Open Babel**	http://openbabel.org

**RDKit**	http://rdkit.org

**Web Applications**	

**ChemDoodle Web Components**	http://web.chemdoodle.com

**Jmol**	http://jmol.org

**Integration**	

**Bioclipse**	http://www.bioclipse.net

**CDK-Taverna**	http://cdktaverna.wordpress.com

**Lensfield2**	https://bitbucket.org/sea36/lensfield2/

**Interconversion**	

**CIFXOM **[95]	https://bitbucket.org/wwmm/cifxom/

**JUMBO-Converters**	https://bitbucket.org/wwmm/jumbo-converters/

**OPSIN**	http://opsin.ch.cam.ac.uk

**OSRA**	http://osra.sf.net

**Structure Databases**	

**Bingo**	http://ggasoftware.com/opensource/bingo

**Chempound (Chem#)**	https://bitbucket.org/chempound

**Mychem**	http://mychem.sf.net

**OrChem**	http://orchem.sf.net

**pgchem**	http://pgfoundry.org/projects/pgchem/

**Text mining**	

**ChemicalTagger **[96]	http://chemicaltagger.ch.cam.ac.uk/

**OSCAR4**	https://bitbucket.org/wwmm/oscar4/

**Computational Chemistry**	

**Avogadro**	http://avogadro.openmolecules.net

**cclib**	http://cclib.sf.net

**GaussSum**	http://gausssum.sf.net

**QMForge**	http://qmforge.sf.net

**Computational Drug Design**	

**Confab **[97]	http://confab.googlecode.com

**Pharao**	http://silicos.be/download

**Piramid**	http://silicos.be/download

**Sieve**	http://silicos.be/download

**Stripper**	http://silicos.be/download

**Other Applications**	

**AMBIT2**	http://ambit.sf.net

**Brunn**	http://brunn.sf.net

**Toxtree**	http://toxtree.sf.net

**XtalOpt**	http://xtalopt.openmolecules.net

### Cheminformatics toolkits

Open Source toolkits for cheminformatics have now existed for nearly ten years. During this period, some toolkits were developed from scratch in academia, whereas others were made Open Source by releasing in-house codebases under liberal licenses. When the Blue Obelisk was established five years ago, the primary toolkits under active development were the Chemistry Development Kit (CDK) [5,6], Open Babel [7], and JOELib [8]. Of these, both the CDK and Open Babel continue to be actively developed. 

The CDK project has been under regular development over the last five years. Several features have been implemented ranging from core components such as an extensible SMARTS matching system and a new graph (and subgraph) isomorphism method [9], to more application oriented components such as 3D pharmacophore searching and matching, and a variety of structural-key and hashed fingerprints. In addition, there have been a number of second generation tools developed on top of the CDK (see below). As well as the use of the CDK in various tools, it has been deployed in the form of web services [10] and has formed the basis of a variety of web applications.

Since 2006, major new features of Open Babel include 3D structure generation and 2D structure-diagram generation, UFF and MMFF94 forcefields, and significantly expanded support for computational chemistry calculations. In addition, a major focus of Open Babel development has been to provide for accurate conversion and representation in areas of stereochemistry, kekulisation, and canonicalisation. The project has also grown, in terms of new contributors, new support from commercial companies, and second-generation tools applying Open Babel to a variety of end-user applications, from molecular editors to chemical database systems.

Two new Open Source cheminformatics toolkits have appeared since the original paper. In 2006 Rational Discovery, a cheminformatics service company (since closed down), released RDKit [11] under the BSD License. This is a C++ library with Python and (more recently) Java bindings. RDKit is actively developed and includes code donated by Novartis. Recent developments include the Java bindings, as well as performance improvements for its database cartridge.

More recently, GGA Software Services (a contract programming company) released the Indigo toolkit [12] and associated software in 2009 under the GPL. Indigo is a C++ library with high-level wrappers in C, Java, Python, and the .NET environment. Like RDKit and other toolkits, Indigo provides support for tetrahedral and cis-trans stereochemistry, 2D coordinate generation, exact/substructure/SMARTS matching, fingerprint generation, and canonical SMILES computation. It also provides some less common functionality, like matching tautomers and resonance substructures, enumeration of subgraphs, finding maximum common substructure of *N *input structures, and enumerating reaction products.

### Second-generation tools

Although feature-rich and robust cheminformatics toolkits are useful in and of themselves, they can also be seen as providing a base layer on which additional tools and applications can be built. This is one of the reasons that cheminformatics toolkits are so important to the open source 'ecosystem'; their availability lowers the barrier for the development of a 'second generation' of chemistry software that no longer needs to concern itself with the low-level details of manipulating chemical structures, and can focus on providing additional functionality and ease-of-use. Although a wide range of chemistry software has been built using Blue Obelisk components (see for example, the "Related Software" link on the Open Babel website, [13] listing over 40 projects as of this writing, or "Software using CDK" at the CDK website), in this section we focus on second-generation tools which themselves have been developed by members of the Blue Obelisk. 

**Figure 1:**
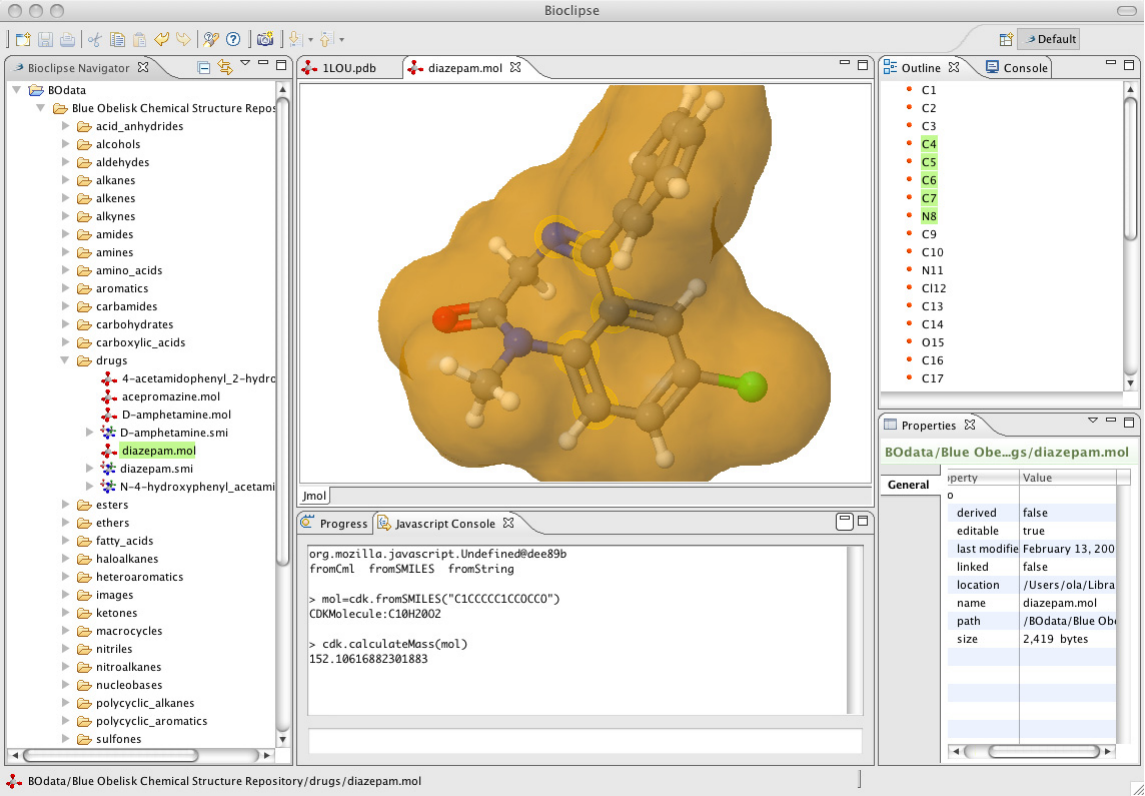
**Screenshot of Bioclipse using Jmol to visualise a molecular surface**.

**Figure 2:**
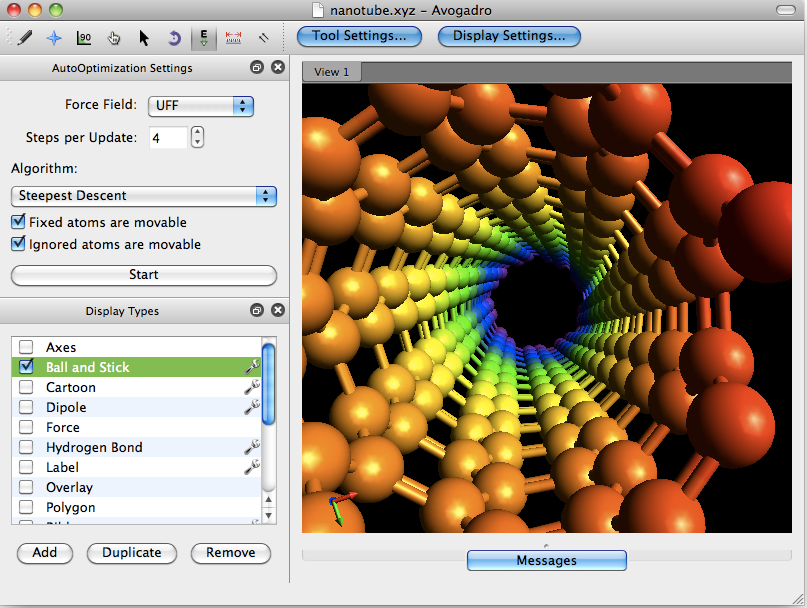
**Screenshot of Avogadro showing a depiction of a carbon nanotube**.

Bioclipse [14] (v2.4 released in Aug 2010) and Avogadro [15] (v1.0 in Oct 2009) are two examples of such software, based on the CDK and Open Babel, respectively. Bioclipse (Figure 1) is an award-winning molecular workbench for life sciences that wraps cheminformatics functionality behind user-friendly interfaces and graphical editors while Avogadro (Figure 2) is a 3D molecular editor and viewer aimed at preparing and analysing computational chemistry calculations. Both projects are designed to be extended or scripted by users through the provision of a plugin architecture and scripting support (using Bioclipse Scripting Language [16], or Python in the case of Avogadro). An interesting aspect of both Avogadro and Bioclipse is that they share some developers with the underlying toolkits and this has driven the development of new features in the CDK and Open Babel.

Both products in turn act as extensible platforms for other software. Bioclipse, for example is used by software such as Brunn [17], a laboratory information system for microplate based high-throughput screening. Brunn provides a graphical interface for handling different plate layouts and dilution series and can automatically generate dose response curves and calculate IC_50_-values. Avogadro is used by Kalzium [18], a periodic table and chemical editor in KDE, and XtalOpt [19,20], an evolutionary algorithm for crystal structure prediction. XtalOpt provides a graphical interface using Avogadro and submits calculations using a range of solid-state simulation software to predict stable polymorphs.

A final example of second-generation Blue Obelisk software is the AMBIT2 [21,22] software, which was developed to facilitate registration of chemicals for the REACH EU directive, and is based on the CDK. It was distributed initially as a standalone Java Swing GUI, and more recently as downloadable web application archive, offering a web services interface to a searchable chemical structures database. Also integrated are descriptor calculations, as well as the ability to run and build predictive models, including modules of the open source Toxtree [22-24] software for toxicity prediction.

### Computational chemistry analysis

Another area where the Blue Obelisk has had a significant impact in the past five years is in supporting quantum chemistry calculations and in interpreting their results. Electronic structure calculations have a long tradition in the chemistry community and a variety of programs exist, mostly proprietary software but with an increasing number of open source codes. However, since each program uses different input formats, and the the output formats vary widely (sometimes even varying between different versions of the same software), preparing calculations and automatically extracting the results is problematic.

Avogadro has already been mentioned as a GUI for preparing calculations. It uses Open Babel to read the output of several electronic structure packages. Avogadro generates input files on the fly in response to user input on forms, as well as allowing inline editing of the files before they are saved to disk. It also features intuitive syntax highlighting for GAMESS input files, allowing expert users to easily spot mistakes before saving an input file to disk.

In addition to this, significant development of new parsing routines took place in an Avogadro plugin to read in basis sets and electronic structure output in order to calculate molecular orbital and electron density grids. This code was written to be parallel, using desktop shared memory parallelism and high level APIs in order to significantly speed up analysis. Most of this code was recently separated from the plugin, and released as a BSD licensed library, OpenQube, which is now used by the latest version of Avogadro. Jmol (see below) can also depict computational chemistry results including molecular orbitals. 

In 2006, the Blue Obelisk project cclib [25] was established with the goal of parsing the output from computational chemistry programs and presenting it in a standard way so that further analyses could be carried out independently of the quantum package used. cclib is a Python library, and the current version (version 1.0.1) supports 8 different computational chemistry codes and extracts over 30 different calculated attributes. Two related Blue Obelisk projects build upon cclib. GaussSum [26], is a GUI that can monitors the progress of SCF and geometry convergences, and can plot predicted UV/Vis absorption and infrared spectra from appropriate logfiles containing energies and oscillator strengths for easy comparison to experimental data. QMForge [27] provides a GUI for various electronic structure analyses such as Frenking's charge decomposition analysis [28] and Mulliken or C-squared analyses on user-defined molecular fragments. QMForge also provides a rudimentary Cartesian coordinate editor allowing molecular structures to be saved via Open Babel.

The Quixote project epitomises the full use of the Blue Obelisk software and is described in detail in another article in this issue. Here we observe that it is possible to convert legacy chemistry file formats of all sorts into semantic chemistry and extract those parts which are suitable for input to computational chemistry programs. This chemistry is then combined with generic concepts of computational chemistry (*e.g*. strategy, machine resources, timing, accuracy etc.) into the legacy inputs for a wide range of programs. Quixote itself follows Blue Obelisk principles in that it does not manage the submission and monitoring of jobs but resumes action when the jobs have been completed, and then applies a range of parsing and transformation tools to create standardised semantic chemical content. A major feature of Quixote is that it requires all concepts to validate against dictionaries and the process of parsing files necessarily generates communally-agreed dictionaries, which represent an important step forward in the Open specifications for Blue Obelisk. When widely-deployed, Quixote will advertise the value of Open community standards for semantics to the world.

The Quixote project is not dependent on any particular technology, other than the representation of computational chemistry in CML and the management of semantics through CML dictionaries. At present, we use JUMBO-Converters [29] for most of the semantic conversion, Lensfield2 [30] for the workflow and Chempound (chem#) [31] to store and disseminate the results.

### Web applications

While desktop software has composed the majority of scientific tools since the computer was introduced, the internet continues to change how applications and content are distributed and presented. The web presents new opportunities for scientists as it is an open and free medium to distribute scientific knowledge, ideas and education. Web applications are software that runs within the browser, typically implemented in Java or JavaScript. Recently, a new version of the HTML specification, HTML5, defined a well-developed framework for creating native web applications in JavaScript and this opens up new possibilities for visualising chemical data.

Jmol, the interactive 3D molecular viewer, is one of the most widely used chemistry applets, and indeed has seen widespread use in other fields such as biology and even mathematics (it is used for 3D depiction of mathematical functions in the Sage Mathematics Projects [32]). It is implemented in Java, and has gone from being a "Rasmol/Chime" replacement to a fully fledged molecular visualisation package, including full support for crystallography [33], display of molecular orbitals from standard basis set/coefficient data, the inclusion of dynamic minimisation using the UFF force field, and a full implementation of Daylight SMILES and SMARTS, with extensions to conformational and biomolecular substructure searching (Jmol BioSMARTS).

**Figure 3:**
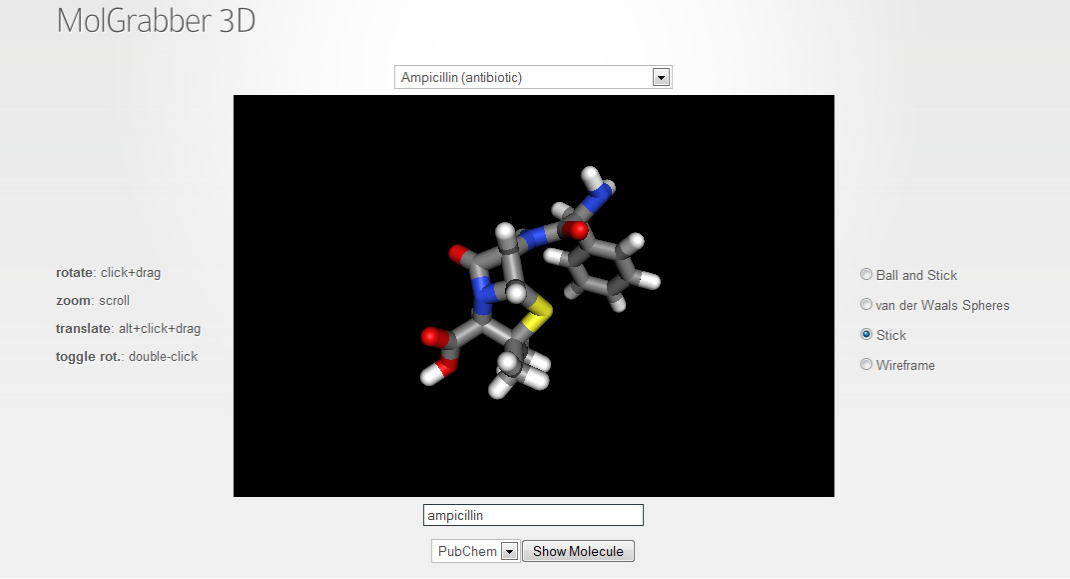
**Screenshot of the MolGrabber 3D demo from ChemDoodle Web Components**.

In 2009, iChemLabs released the ChemDoodle Web Components library [34] under the GPL v3 license (with a liberal HTML exception). This library is completely implemented in JavaScript and uses HTML5 to allow the scientist to present publication quality 2D and 3D graphics (see Figure 3) and animations for chemical structures, reactions and spectra. Beyond graphics, this tool provides a framework for user interaction to create dynamic applications through web browsers, desktop platforms and mobile devices such as the iPhone, iPad and Android devices.

### The business end

Open Source provides a unique opportunity for commercial organisations to work with the cheminformatics community. Traditional business models rely on monetisation of source code, causing companies to repeat work done by other companies. This model is sometimes combined with a free (gratis) model for people working at academic institutes, to increase adoption and encourage contributions from academics. This solution defines the return on investment as the IP on the software, but has the downside of investment losses due to duplication of software and method development, which become visible when proprietary companies merge. Some authors have argued that in the chemistry field few contributors are available to volunteer time to improve codes and IP considerations may prevent contributions from industry [35]. If true, this would hamper adoption of Open Source and Open Data in chemistry, and greatly slow the growth of projects such as those in the Blue Obelisk.

The Blue Obelisk community, however, takes advantage of the fact that much of the investment needed for development is either paid for by academic institutes and funding schemes, or by volunteers investing time and effort. In return, contributors get full access to the source code, and the Open Source licensing ensures that they will have access any time in the future. In this way, the license functions as a social contract between everyone to arrange an immediate return on investment. Effiectively, this approach shares the burden of the high investment in having to develop cheminformatics software from scratch, allowing researchers and commercial partners alike to focus on their core business, rather than the development of prerequisites. In the case of the Blue Obelisk, the rich collection of Open Source cheminformatics tools provided greatly reduces investment up front for new companies in the cheminformatics market. Such advantages have also been noted in the drug discovery field [36-38].

The use of Open Standards allows everyone to select those Blue Obelisk components they find most useful, as they can easily replace one component with another providing the same functionality, taking advantage that they use the same standards for, for example, data exchange. This way, licensing issues are becoming a marginal problem, allowing companies to select a license appropriate for their business model. This too, allows a company to create a successful product with significantly reduced cost and effort.

At the time of writing there are many commercial companies developing chemistry solutions around Open Source cheminformatics components provided by the Blue Obelisk community. Examples of such companies include iChemLabs, IdeaConsult, Wingu, Silicos, GenettaSoft, eMolecules, hBar, Metamolecular, and Inkspot Science. Some of these merely use components, but several actively contribute back to the Blue Obelisk project they use, or donate new Open Source cheminformatics projects to the community. 

For example, iChemLabs released the ChemDoodle Web Components library under the GPL v3 license, based on the upcoming HTML5 Open Standard. It allows making web and mobile interfaces for chemical content. The project is already being adopted by others, including iBabel [39], ChemSpotlight [40] and the RSC ChemSpider [41,42].

Silicos has released several Open Source utilities [43] based on Open Babel, such as Pharao, a tool for pharmacophore searching, Sieve for filtering molecular structure by molecular property, Stripper for removing core scaffold structures from a molecule set, and Piramid for molecular alignment using shape determined by the Gaussian volumes as a descriptor. Additionally, contributions have been made to the Open Babel project itself.

Other companies use Blue Obelisk components and contribute patches, smaller and larger. For example, IXELIS donated the isomorphism code in the CDK, eMolecules donated canonicalisation code to Open Babel, Metamolecular improved the extensibility and unit testing suite of OPSIN, and AstraZeneca contributed code to the CDK for signatures. This is just a very minor selection, and the reader is encouraged to contact the individual Blue Obelisk projects for a detailed list.

In May 2011, a Wellcome Trust Workshop on Molecular Informatics Open Source Software (MIOSS) explored the role of Open Source in industrial laboratories and companies as well as academia (several of the presenters are among the authors of this paper). The meeting identified that Open Source software was extremely valuable to industry not just because it is available for free, but because it allows the validation of source code, data and computational procedures. Some of the discussion was on business models or other ways to maintain development of Open Source software on which a business relied. Companies are concerned about training and support and, in some cases, product liability. There are difficulties for software for which there is no formal transaction other than downloading and agreeing to license terms. One anecdote concerned a company that wished to donate money to an Open Source project but could not find a mechanism to do so.

Industry participants also pointed out that there is a considerable amount of contribution-in-kind from industry, both from enhancements to software and also the development of completely new software and toolkits. Companies are now finding it easier to create mechanisms for releasing Open Source software without violating confidentiality or incurring liability. A phrase from the meeting summed it up: "The ice is beginning to melt", signifying that we can expect a rapid increase in industry's interest in Open Source.

### Converting chemical names and images to structures

The majority of chemical information is not stored in machine-readable formats, but rather as chemical names or depictions. The OSRA and OPSIN projects focus on extracting chemical information from these sources. Such software plays a particularly important role for data mining the chemical literature, including patents and theses.

Optical Structure Recognition Application (OSRA) [44] was started in early 2007 with the goal to create the first free and open source tool for extraction and conversion of molecular images into SMILES and SD files. From the very beginning the underlying philosophy was to integrate existing open source libraries and to avoid "reinventing the wheel" wherever possible. OSRA relies on a variety of open source components: Open Babel for chemical format conversion and molecular property calculations, GraphicsMagick for image manipulation, Potrace for vectorisation, GOCR and OCRAD for optical character recognition. The growing importance of image recognition technology can be seen in the fact that only a few years ago there was only one widely available software package for chemical structure recognition - CLiDE (commercially developed at Keymodule, Ltd), but today there are as many as seven available programs.

OPSIN (Open Parser for Systematic IUPAC Nomenclature) [45] focuses instead on interpreting chemical names. The chemical name is the oldest form of communication used to describe chemicals, predating even the knowledge of the atomic structure of compounds. Chemical names are abundant in the scientific literature and encode valuable structural information. Through successive books of recommendations [46,47], IUPAC has tried to codify and to an extent standardise naming practices. OPSIN aims to make this abundance of chemical names machine readable by translating them to SMILES, CML or InChI. The program is based around the use of a regular grammar to guide tokenisation and parsing of chemical names, followed by step-wise application of nomenclature rules. It is able to offer fast and precise conversions for the majority of names using IUPAC organic nomenclature, and is available as a web service, Java library and standalone application for maximum interoperability.

### Chemical database software

Registration, indexing and searching of chemical structures in relational databases is one of the core areas of cheminformatics. A number of structure registration systems have been published in the last five years, exploiting the fact that Open Source cheminformatics toolkits such as Open Babel and the CDK are available. OrChem [48], for example, is an open source extension for the Oracle 11G database that adds registration and indexing of chemical structures to support fast substructure and similarity searching. The cheminformatics functionality is provided by the CDK. OrChem provides similarity searching with response times in the order of seconds for databases with millions of compounds, depending on a given similarity cut-off. For substructure searching, it can make use of multiple processor cores on today's powerful database servers to provide fast response times in equally large data sets.

Besides the traditional and proven relational database approach with added chemical features ('cartridges'), there is growing interest in tools and approaches based on the web philosophy and practice. Several groups [49,50] are experimenting with the Resource Description Framework (RDF) language on the assumption that generic high-performance solutions will appear. RDF allows everything to be described by URIs (data, molecules, dictionaries, relations). The Chempound system [31], as deployed in Quixote and elsewhere, is an RDF-based approach to chemical structures and compounds and their properties. For small to medium-sized collections (such as an individual's calculations or literature retrieval), there are many RDF tools (e.g. SIMILE, Apache Jena) which can operate in machine memory and provide the flexibility that RDF offers. For larger systems, it is unclear whether complete RDF solutions (e.g. Virtuoso) will be satisfactory or whether a hybrid system based on name-value pairs (e.g. CouchDB, MongoDB) will be sufficient.

### Collaboration and interoperability

**Figure 4:**
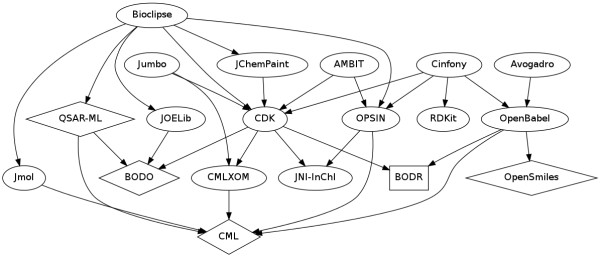
**Dependency diagram of some Blue Obelisk projects**. Each block represents a project. Square blocks show Open Data, ovals are Open Source, and diamonds are Open Standards.

One of the successes of the Blue Obelisk has been to bring developers together from different Open Source chemistry projects so that they look for opportunities to collaborate rather than compete, and to leverage work done by other projects to avoid duplication of effort. As an example of this, when in March 2008 the Jmol development team were looking to add support for energy minimisation, rather than implement a forcefield from scratch they ported the UFF forcefield [51] implementation from Open Babel to Jmol. This code enables Jmol to support 2D to 3D conversion of structures (through energy minimisation). In a similar manner, efficient Jmol code for atom-atom rebonding has been ported to the CDK. Figure 4 shows the collaborative nature of software developed in the Blue Obelisk, as one project builds on functionality provided by another project.

Another collaborative initiative between Blue Obelisk projects was the establishment in May 2008 of the ChemiSQL project. This brought together the developers of several open source chemistry database cartridges (PgChem [52], Mychem [53], OrChem [48] and more recently Bingo [54]) with a view to making their database APIs more similar and collaborating on benchmark datasets for assessing performance. For two of these projects, PgChem and Mychem, which are both based on Open Babel, there is the additional possibility of working together on a shared codebase.

In the area of cheminformatics toolkits, two of the existing toolkits Open Babel and RDKit are planning to work together on a common underlying framework called MolCore [55]. This project is still in the planning stage, but if it is a success it will mean that the the two libraries will be interoperable (while retaining their existing focus) but also that the cost of maintaining the code will be shared among more developers, freeing time for the development of new features.

One of the goals of the Blue Obelisk is to promote interoperability in chemical informatics. When barriers exist to moving chemical data between different software, the community becomes fragmented and there is the danger of vendor lock-in (where users are constrained to using a particular software, a situation which puts them at a disadvantage). This applies as much to Open Source software as to proprietary software. Cinfony is a project (first release in May 2008) whose goal is to tackle this problem in the area of cheminformatics toolkits [56]. It is a Python library that enables Open Babel, the CDK, and RDKit (and shortly, Indigo and OPSIN) to be used using the same API; this makes it easy, for example, to read a molecule using Open Babel, calculate descriptors using the CDK and create a depiction using RDKit. 

Another way through which interoperability of Blue Obelisk projects has been promoted and developed is through integration into workflow software such as Taverna [57] and KNIME [58] (both open source). Such software makes it easy to automate recurring tasks, and to combine analyses or data from a variety of different software and web services. A combination of the Chemistry Development Kit and Taverna, for instance, was reported in 2010 [59]. In the case of KNIME, it comes with built-in basic collection of CDK-based and Open Babel-based nodes, while other nodes for the RDKit and Indigo are available from KNIME's "Community Updates" site.

## Open Standards

### Chemical Markup Language, CML

Chemical Markup Language (CML) is discussed in several articles in this issue, and a brief summary here re-iterates that it is designed primarily to create a validatable semantic representation for chemical objects. The five main areas (molecules, reactions, computational chemistry, spectra and solid-state (see above)) have now all been extensively deployed and tested. CML can therefore be used as a reference for input and output for Blue Obelisk software and a means of representing data in Blue Obelisk resources.

CML, being an XML application, can inter-operate with other markup languages and in particular XHTML, SVG, MathML, docx and more specialised applications such as UnitsML and GML (geosciences). We believe that it would be possible using these languages to encode large parts of, say, first year chemistry text books in XML. Similarly, it is possible to create compound documents with word processing or spreadsheet software that have inter-operating text, graphics and chemistry (as in Chem4Word). Being a markup language, CML is designed for re-purposing, including styling, and therefore a mixture of these languages can be used for chemical catalogues, general publications, logbooks and many other types of document in the scientific process.

CML describes much of its semantics through conventions and dictionaries, and the emerging ecosystem (especially in computational chemistry) is available as a semantic resource for many of the applications and specifications in this article.

### InChI

The IUPAC InChI identifier is a non-proprietary and unique identifier for chemical substances designed to enable linking of diverse data compilations. Prior to the development of the InChI identifier chemical information systems and databases used a wide variety of (generally proprietary) identifiers, greatly limiting their interoperability. Although its development predates the Blue Obelisk, software such as Open Babel has included InChI support since 2005, and support for InChI in Indigo is due in 2011.

Since the official InChI implementation is in C, it is difficult to access from the other widely used language for cheminformatics toolkits, Java. Early attempts to generate InChI identifiers from within Java involved programatically launching the InChI executable and capturing the output, an approach that was found to be fairly unreliable and broke the 'write once, run anywhere' philosophy of Java. The Blue Obelisk project JNI-InChI [60] was established in 2006 to solve this problem by using the Java Native Interface framework to provide transparent access to the InChI library from within Java and other Java Virtual Machine (JVM) based languages, supporting the wider adoption of this standard identifier by the chemistry community. 

The Java Native Interface framework provides a mechanism for code running inside the JVM, to place calls to libraries written in languages such as C, C++ and Fortran, and compiled into native, machine specific, code. JNI-InChI provides a thin C wrapper, with corresponding Java code, around the IUPAC InChI library, exposing the InChI library's functionality to the JVM. To overcome the need to have the correct InChI library pre-installed on a system, JNI-InChI comes with a variety of precompiled native binaries and automatically extracts and deploys the correct one for the detected operating system and architecture. The JNI-InChI library comes with native binaries supporting a range of operating systems and architectures; the current version has binaries for 32- and 64-bit Windows, Linux and Solaris, 64-bit FreeBSD and 64-bit Intel-based Mac OS X - a number of which are not supported by the original IUPAC distribution of InChI. The JNI-InChI project has matured to support the full range of functionality of the InChI C library: structure-to-InChI, InChI-to-structure, AuxInfo-to-structure, InChIKey generation, and InChI and InChIKey validation. JNI-InChI provides the InChI functionality for a number of Open Source projects, including the Chemistry Development Kit, Bioclipse and CMLXOM/JUMBO, and is also used by commercial applications and internally in a number of companies. Through its widespread use and Open Source development model, a number of issues in earlier versions of the software have been identified and resolved, and JNI-InChI now offers a robust tool for working with InChIs in the JVM.

### OpenSMILES

One of the most widely used ways to store chemical structures is the SMILES format (or SMILES string). This is a linear notation developed by Daylight Information Systems that describes the connection table of a molecule and may optionally encode chirality. Its popularity stems from the fact that it is a compact representation of the chemical structure that is human readable and writable, and is convenient to manipulate (e.g. to include in spreadsheets, or copy from a web page).

Despite its widespread use, a formal definition of the language did not exist beyond Daylight's SMILES Theory Manual and tutorials. This caused some confusion in the implementation and interpretation of corner cases, for example the handling of cis/trans bond symbols at ring closures. In 2007, Craig James (eMolecules) initiated work on the OpenSMILES specification [61], a complete specification of the SMILES language as an Open Standard developed through a community process. The specification is largely complete and contains guidelines on reading SMILES, a formal grammar, recommendations on standard forms when writing SMILES, as well as proposed extensions.

### QSAR-ML

The field of QSAR has long been hampered by the lack of open standards, which makes it difficult to share and reproduce descriptor calculations and analyses. QSAR-ML was recently proposed as an open standard for exchanging QSAR datasets [62]. A dataset in QSAR-ML includes the chemical structures (preferably described in CML) with InChI to protect integrity, chemical descriptors linked to the Blue Obelisk Descriptor Ontology [63], response values, units, and versioned descriptor implementations to allow descriptors from different software to be integrated into the same calculation. Hence, a dataset described in QSAR-ML is completely reproducible. To allow for easy setup of QSAR-ML compliant datasets, a plugin for Bioclipse was created with a graphical interface for setting up QSAR datasets and performing calculations. Descriptor implementations are available from the CDK and JOELib, as well as via remote web services such as XMPP [64].

### Remaining challenges

A core requirement for chemical structure databases and chemical registration systems in general is the notion of structure standardisation. That is, for a given input structure, multiple representations should be converted to one canonical form. Structure canonicalisation routines partially address this aspect, converting multiple alternative topologies to a single canonical form. However, the problem of standardisation is broader than just topological canonicalisation. Features that must be considered include

• topological canonicalisation

• handling of charges

• tautomer enumeration and canonicalisation

• normalisation of functional groups

Currently, most of the individual components of a 'standardisation pipeline' can be implemented using Blue Obelisk tools. The larger problem is that there is no agreed upon list of steps for a standardisation process. While some specifications have been published (e.g., PubChem) and some standardisation services and tools are available (for example, PubChem provides an online service to standardise molecules [65]) each group has their own set of rules. A common reference specification for standardisation would be of immense value in interoperability between structure repositories as well as between toolkits (though the latter is still confounded by differences in lower level cheminformatic features such as aromaticity models). 

We have already discussed the development of an Open SMILES standard. While much progress has been made towards a complete specification, more remains to be done before this can be considered finished. After that point, the next logical step would be to start work on a standard for the SMARTS language, the extension to SMILES that specifies patterns that match chemical substructures.

## Open Data

A considerable stumbling block in advocating the release of scientific data as Open Data has been how exactly to define "Open." A major step forward was the launch in 2010 of the Panton Principles for Open Data in Science [66]. This formalises the idea that Open Data maximises the possibility of reuse and repurposing, the fundamental basis of how science works. These principles recommend that published data be licensed explicitly, and preferably under CC0 (Creative Commons 'No Rights Reserved', also known as CCZero) [67]. This license allows others to use the data for any purpose whatsoever without any barriers. Other licenses compatible with the Panton Principles include the Open Data Commons Public Domain Dedication and Licence (PDDL), the Open Data Commons Attribution License, and the Open Data Commons Open Database License (ODbL) [68].

Despite this positive news, little chemical data compatible with these principles has become available from the traditional chemistry fields of organic, inorganic, and solid state chemistry. Table 2 lists a few notable exceptions, some of which are discussed further below. There is also data available using licenses not compatible with the Panton Principles, but where the user is allowed to modify and redistribute the data. A new data set in this category is the data from the ChEMBL database [69], which is available under the Creative Commons Share-Alike Attribution license. The RSC ChemSpider database [41], although not fully Open, also hosts Open Data; for example, spectral data when deposited can be marked as Open. 

Importantly, publishing data as CC0 is becoming easier now that websites are becoming available to simplify publishing data. Two projects that can be mentioned in this context are FigShare [70], where the data behind unpublished figures can be hosted, and Dryad [71] where data behind publications can be hosted. Initiatives like this make it possible to host small amounts of data, and those combined are expected to become soon a substantial knowledge base.

**Table 2:** Open Data in chemistry.

Name	License/Waiver	Description
**Chempedia **[98]	CC0	Crowd-sourced chemical names (project discontinued but data still available)

**CrystalEye **	PPDL	Crystal structures from primary literature

**ONS Solubility **	CC0	Solubility data for various solvents

**Reaction Attempts **	CC0	Data on successful and unsuccessful reactions

Overview of major open chemical data available under a license or waiver compatible with the Panton Principles.

### Reaction Attempts

Although there are existing databases that allow for searching reactions, those using Open Data are harder to find. The Reaction Attempts database [72], to which anyone can submit reaction attempts data, consists mainly of reaction information abstracted from Open Notebooks in organic chemistry, such as the UsefulChem project from the Bradley group [73] and the notebooks from the Todd group [74]. Key information from each experiment is abstracted manually, with the only required information consisting of the ChemSpider IDs of the reactants and the product targeted in the experiment; and a link to the laboratory notebook page. Information in the database can be searched and accessed using the web-based Reaction Attempts Explorer [75].

Since the database reflects all data from the notebooks, it includes experiments in progress, ambiguous results and failed runs. Unlike most reaction databases that only identify experiments successfully reported in the literature, the Reaction Attempts Explorer allows researchers to easily find patterns in reactions that have already been performed, and since the data are open and results are reported across all research groups, intersections are easily discovered and possible Open Collaboration opportunities are easily found [76,77].

### Non-Aqueous Solubility

Although the aqueous solubility of many common organic compounds is generally available, quantitative reports of non-aqueous solubility are more difficult to find. Such information can be valuable for selecting solvents for reactions, re-crystallization and related processes. In 2008, the Open Notebook Science Solubility Challenge was launched for the purpose of measuring non-aqueous solubility of organic compounds, reporting all the details of the experiments in an Open Notebook and recording the results as Open Data in a centralized database [78,79]. This crowdsourcing project was also supported by Submeta, Sigma-Aldrich, Nature Publishing Group and the Royal Society of Chemistry. The database currently holds 1932 total measurements and 1428 averaged solute/solvent measurements all of which are available under a CC0 license. Several web services and feeds are available to filter and re-use the dataset [80]. In particular, models have been developed for the prediction of non-aqueous solubility in 72 different solvents [81] using the method of Abraham et al [82] with descriptors calculated by the Chemistry Development Kit. These models are available online and will be refined as more solubility data is collected.

### The Blue Obelisk Data Repository (BODR)

The Blue Obelisk has created a repository of key chemical data in a machine-readable format [83]. The BODR focuses on data that is commonly required for chemistry software, and where there is a need to ensure that values are standard between codes. Examples are atomic masses and conversions between physical constants. These data can be used by others for any purpose (for example, for entry into Wikipedia or use in in-house software), and should lead to an enhancement in the quality of community reference data. The Blue Obelisk provides also a complementary project, the Chemical Structure Repository [83]. It aims to provide 3D coordinates, InChIs and several physico-chemical descriptors for a set of 570 organic compounds.

### NMRShiftDB

NMRShiftDB [84,85] represents one of the earliest resources for Open community-contributed data (first released in 2003). Research groups that measure NMR spectra or extract it from the literature can contribute that information to NMRShiftDB which provides an Open resource where entries can be searched by chemical structure or properties (especially peaks). Although it is difficult to encourage large amounts of altruistic contribution (as happens with Wikipedia), an alternative possible source of data could come from linking data capture with data publication. For example, the Blue Obelisk has enough software that it is possible to create a seamless chain for converting NMR structures in-house into NMRShiftDB entries. If and when the chemistry community encourages or requires semantic publication of spectra rather than PDFs, it would be possible to populate NMRShiftDB rapidly along the the lines of CrystalEye (see below). A similar approach has been demonstrated earlier using the Blue Obelisk components Oscar and Bioclipse using text mining approaches [86].

### CrystalEye

CrystalEye [87] is an example of cost-effiective extraction of data from the literature where this is published both Openly and semantically. Software extracts Openly-published crystal structures from a variety of scholarly journals, processes them and then makes them available through a web interface. It currently contains about 250,000 structures. CrystalEye serves as a model for a high-value, high-quality Open data resource, including the licensing of each component as Panton-compatible Open data.

## Other areas of activity

**Figure 5:**
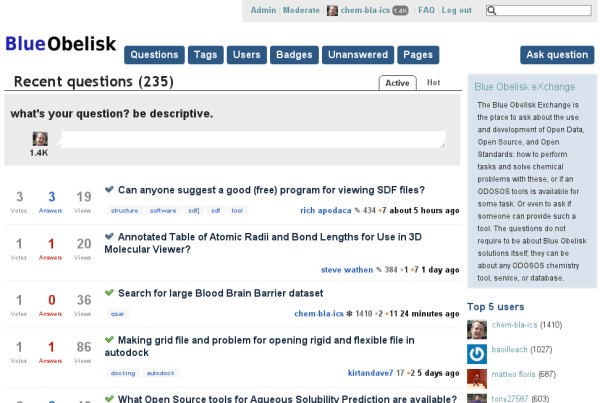
**Screenshot of the Blue Obelisk eXchange Question and Answer website**.

While each Blue Obelisk project has its own website and point of contact (typically a mailing list), because of the breadth of Blue Obelisk projects it can be difficult for a newcomer to understand which of them, if any, can best address a particular problem. To address this issue, members of the Blue Obelisk established a Question & Answer website [88] (see Figure 5). This is a website in the style of Stack Overflow [89] that encourages high quality answers (and questions) through the use of a voting system. In the year since it was established, over 200 users have registered, many of whom had no previous involvement with the Blue Obelisk, showing that the Q&A website complements earlier existing channels of communication.

The rise of self-publishing and print-on-demand services has meant that publishing a book is now as straightforward as uploading to an appropriate website. Unlike the traditional publishing route where books with projected low sales volume would be expensive, websites such as Lulu [90] allow the sale of low-priced books on chemistry software, and books are now available for purchase on Jmol [91], the Chemistry Development Kit [92] and Open Babel [93].

## Conclusions

We have shown that the Blue Obelisk has been very successful in bringing together researchers and developers with common interests in ODOSOS, leading to development of many useful resources freely available to the chemistry community. However, how best to engage with the wider chemistry community outside of the Blue Obelisk remains an open question. If the Blue Obelisk is truly to make an impact, then an attempt must be made to reach beyond the subscribers to the Blue Obelisk mailing list and blogs of members.

We hope to see this involvement between the Blue Obelisk and the wider community grow in the future. To this end, we encourage the reader to visit the Blue Obelisk website [94], send a message to our mailing list, investigate related projects or read our blogs.

## Competing interests

The authors declare that they have no competing interests.

## Authors' contributions

The overall layout of the manuscript grew from discussions between NMOB, RG and ELW. The authorship of the paper is drawn from those people connected with fully Open Data/Standards/Source (OSI-compliant or OKF-compliant) projects associated with the Blue Obelisk. There are a large number of people contributing to these projects and because those projects are published in their own right it is not appropriate to include all their developers by default. We invited a number of 'project gurus' who have been active in promoting the Blue Obelisk, to be authors on this paper and most have accepted and contributed.
